# Effects of Intra- and Interchain Interactions on Exciton Dynamics of PTB7 Revealed by Model Oligomers

**DOI:** 10.3390/molecules25102441

**Published:** 2020-05-23

**Authors:** Thomas J. Fauvell, Zhengxu Cai, Matthew S. Kirschner, Waleed Helweh, Pyosang Kim, Tianyue Zheng, Richard D. Schaller, Luping Yu, Lin X. Chen

**Affiliations:** 1 Department of Chemistry, Northwestern University, 2145 Sheridan Road, Evanston, IL 60208, USA; tfauvell@gmail.com (T.J.F.); kirschner.21@gmail.com (M.S.K.); waleedhelweh2022@u.northwestern.edu (W.H.); pyosang.kim@northwestern.edu (P.K.); schaller@anl.gov (R.D.S.); 2 LEAP Center, Northwestern University, 2145 Sheridan Road, Evanston, IL 60208, USA; 3 Chemical Sciences and Engineering Division, Argonne National Laboratory, 9700 South Cass Avenue, Argonne, IL 60439, USA; tyzheng258@gmail.com; 4 Department of Chemistry and James Frank Institute, The University of Chicago, 929 East 57th Street, Chicago, IL 60637, USA; caizx@bit.edu.cn

**Keywords:** PTB7, OPV, excited state, oligomer, structural dynamics, electronic processes

## Abstract

Recent studies have shown that molecular aggregation structures in precursor solutions of organic photovoltaic (OPV) polymers have substantial influence on polymer film morphology, exciton and charge carrier transport dynamics, and hence, the resultant device performance. To distinguish photophysical impacts due to increasing π-conjugation from chain lengthening and π–π stacking from single/multi chain aggregation in solution and film, we used oligomers of a well-studied charge transfer polymer PTB7 with different lengths as models to reveal intrinsic photophysical properties of a conjugated segment in the absence of inter-segment aggregation. In comparison with previously studied photophysical properties in polymeric PTB7, we found that oligomer dynamics are dominated by a process of planarization of the conjugated backbone into a quinoidal structure that resembles the self-folded polymer and that, when its emission is isolated, this quinoidal excited state resembling the planar polymer chain exhibits substantial charge transfer character via solvent-dependent emission shifts. Furthermore, the oligomers distinctly lack the long-lived charge separated species characteristic of PTB7, suggesting that the progression from charge transfer character in isolated chains to exciton splitting in neat polymer solution is modulated by the interchain interactions enabled by self-folding.

## 1. Introduction

Organic Photovoltaics (OPVs) offer affordable, lightweight, and flexible solar energy harvesting with low energy payback times [1], making them important devices in the transition into renewable energy. Recent advances in device technology, with efficiencies for tandem cells eclipsing 17% [2,3,4], have demonstrated the technology’s viability and promises in solar energy utilization. Substantial progress on understanding the structural influence on electronic properties has been made for the conjugated polymers and small molecules that comprise OPV active layers, but it is still challenging relating these structural characteristics to optoelectronic functions in complex and significantly disordered assemblies, such as their aggregates in solution and film domains. A recent study correlated a variety of polymer properties—optical gap, charge separation driving force, short circuit current (J_sc_), open circuit voltage (V_oc_), fill factor (FF), and electronic transition dipoles, with devices PCEs among 150 polymers [5]. Surprisingly, the results suggested that device parameters related to charge transport and extraction (J_sc_ and FF), and, hence, film morphology, are key for high performance OPV devices. Moreover, other recent works on high performing OPV materials indicate that polymer preassembly in solution via aggregation is important for producing film morphology that facilitates high OPV performance, with efforts in understanding and utilizing these effects resulting in high device efficiencies [3,6,7,8]. Since manipulating solution aggregation appears to be a useful strategy in the pursuit of higher device efficiency, understanding its effects on both the resultant film morphology and optical characteristics is of the utmost importance.

Because of its broad absorption and low bandgap, efficient exciton dissociation, favorable film morphology, and high performance, **PTB7** (a monofluorinated polybenzodithiophene-thienothiophene polymer or Poly({4,8-bis[(2-ethylhexyl)oxy]benzo[1,2-b:4,5-b′]dithiophene-2,6-diyl}{3-fluoro-2-[(2-ethylhexyl)carbonyl]thieno[3,4-b]thiophenediyl})) has been a stalwart of OPV research for years and served as inspiration for many newer high performing OPV polymers [9,10,11,12,13,14]. Therefore, it is of interest to investigate unique properties of this polymer relevant to its function. Unlike previously studied conjugated polymers with dramatic optical spectral shifts from solution to film to annealed film [15,16,17,18], **PTB7** has nearly identical spectra independent of the media, which inspired our recent work that has highlighted the beneficial effects of **PTB7′**s unique solution self-folding and its resulting HJ aggregate [18,19,20] energetic structure.

**Figure 1:**
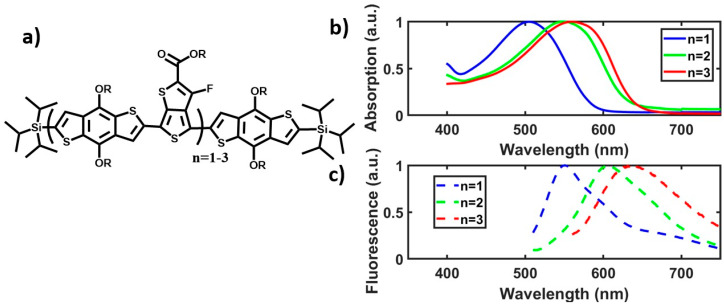
(**a**) Structure of (BDT-TT)_n_BDT **PTB7** oligomer series. The R group is 2-ethylhexyl. Molecules are capped by TIPS (tri-isopropylsilyl) groups. (**b**) Normalized absorption spectra of n = 1–3 **PTB7** oligomer series. The broad, featureless curves show a large shift between n = 1 and n = 2, but less of a shift between n = 2 and n = 3. (**c**) Normalized fluorescence spectra of n = 1–3 oligomers. The peak shift of the maxima does not exactly follow those in the absorption. There is some structure, with shoulders especially in n = 1.

In that work [20], a series of oligomers with the alternating benzodithiophene and fluorinated thienothiophene ((BDT-TT)_n_BDT, n = 1–3) backbone structure of **PTB7** (structure in Figure 1a) demonstrated backbone length-dependent steady state optical absorption and emission properties. The distinct vibronic features clearly observed in **PTB7′**s absorption spectra were absent in the oligomers. Instead, a broad featureless absorption band was observed for each oligomer up to n = 3. Although the absorption peak position red-shifts as a function of n, it reaches to a saturation at n = 3, at which the peak maximum was still > 3000 cm−1 higher in energy than that of **PTB7**. Accompanying this peak maxima shift was a transformation in line shape from a broad featureless band (n = 1–3) to a much red-shifted peak with clear vibronic features in those with intermediate length (n = 7–40) and the polymer (n > 40) spectra. These results, although puzzling initially, can be explained by a transformation from the conformationally diverse short oligomers to self-folded polymers [20]. It was deduced that **PTB7** self-folds in solution, resulting in interchain π–π stacking, planarizing the polymer backbone and enabling intrachain charge transfer. While previous work had attributed the polymer’s red-shifted absorption, a key contributor to its high efficiency [21] exclusively to its alternating electron-rich and electron-poor (“push–pull”) backbone structure [22,23,24]. It was found that there is synergistic contribution from the enhanced planarization and excitonic coupling created by the HJ aggregate nature induced by the polymer’s self-folding. This robust self-folding of the polymer in solution explained persistent spectral features in both dilute solution and film, mid-gap fluorescence spectrum, and apparent order in disordered films.

**PTB7** in dilute solution has been shown to have spectral signatures of a long-lived “Charge Separated” (CS) state, a precursor to free charge carriers, which was previously observed only in BHJ blends with electron acceptors such as **PCBM** (Phenyl-C_61_-buyric acid methyl ester) [22,25] in ultrafast experiments. This observation was initially attributed to the intrinsic charge transfer character along the polymer backbone due to alternating electron donating and withdrawing blocks: the local differences in electron affinity would induce a spatial displacement of hole and electron in the HOMO-LUMO transition. These polarized excitons would have defrayed binding energy, enabling intrachain exciton splitting to yield the CS state. However, these studies assumed that **PTB7** were isolated chains in solution [22,25], which was subsequently proven otherwise [20], because the self- folding found in our later study would enable both intrachain and interchain interactions even in dilute solution.

Although our previous study [20] proved the self-folding in the polymer **PTB7** and its absence in short oligomers in solution, the intra- and interchain interactions on the exciton pathways and dynamics was not studied. In this study, we use these **PTB7** oligomers as a model series to probe exciton delocalization and structural dynamics in unfolded polymer segments without their inherent self-folding in order to distinguish effects of intra- and interchain interactions on the exciton dynamics. Although these oligomers have only a few repeating units of BDT-TT, they have fully extended lengths of 18.7, 30.9, and 43.2 Å (excluding TIPS capping groups), making them useful analogues of unfolded polymer segments. Specifically, the emission spectrum of the n = 2 oligomer coincides with mid-gap fluorescence exhibited by the polymer [20], echoing previous work with **P3HT** (poly(3- hexylthiophene-2,5-diyl)) where emission comparisons revealed an exciton of similar size to hexathiophene [26]. The study of the excited state dynamics of the **PTB7** oligomer series will serve as a basis for understanding length-dependent photophysical behaviors in the absence of polymeric solution folding and allow the separation of contributions from **PTB7′**s repeating structure and robust folding on its exciton dynamics and charge transfer character.

## 2. Results and Discussions

### 2.1. Excited State and Structural Dynamics in Oligomers

In order to discuss the ultrafast dynamics below, structures of the **PTB7** oligomers are shown in Figure 1a, along with their steady state absorption and fluorescence spectra in Figure 1b–c, respectively. As demonstrated previously [20], the length-dependent absorption maxima red-shifts as n increases, but the shift saturates at n > 3 due to conformational disorder concerning the C-C bonds connecting adjacent repeating units, which attenuates the π-conjugation along the backbone [27]. It is worth noting, however, the fluorescence spectra in Figure 1c do not strictly follow this pattern and further shifts could happen in oligomers with n > 3 following the observed trend. Such differences in the trend of red-shift may reflect conformational differences between the absorbing and emission species for these oligomers in terms of π-conjugation lengths for the ground and excited species.

**Figure 2:**
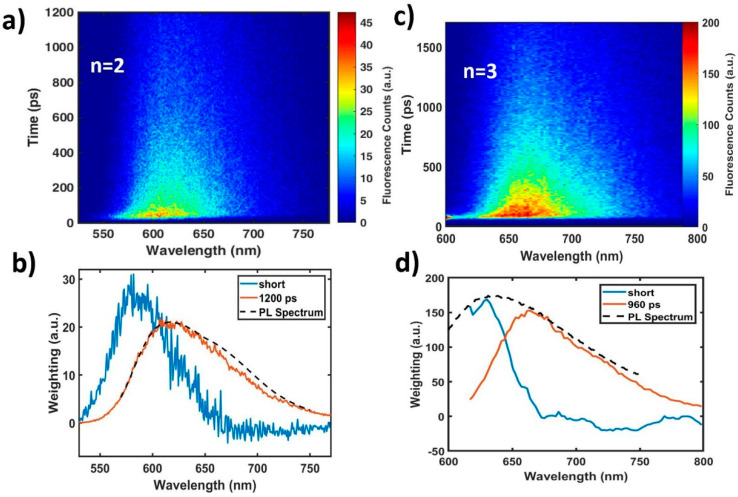
(**a**) Streak camera time-resolved fluorescence measurements of the n = 2 oligomer at 500 nm excitation. The time constant for the short-lived (blue line), blue-shifted fluorescent species is limited by the time resolution of the apparatus (Instrument response function FWHM ~20 ps). (**b**) Decay-associated spectra of streak camera data in (**a**). The blue-shifted component’s lifetime was too short to resolve. The steady state photoluminescence spectrum (dashed line) is scaled for comparison. (**c**) Same measurements as (**a**) for the n = 3 oligomer at 550 nm excitation, which show similar emission characteristics as n = 2 but considerably red-shifted in both short and longer-lived fluorescence peaks. (**d**) Decay-associated spectra of streak camera data for n = 3. The steady state photoluminescence spectrum is scaled arbitrarily for clarity.

To further investigate, we performed time-resolved fluorescence measurements using a streak camera and the results are shown in Figure 2. The n = 1 oligomer was unstable under the laser excitation and its results are shown in the SI (Figures S16 and S17, Tables S6 and S7). The time-resolved fluorescence spectra for n = 2 and n = 3 oligomers are shown respectively in Figure 2a–c. Both contain fast spectral evolution on the blue edge of the spectrum and longer-lived fluorescence at the red edge of the spectrum, which are represented in the two-component decay-associated spectrum in Figure 2b,d (see Methods and SI for fitting details) from the global fitting of the data. Beginning with the n = 2 oligomer in Figure 2b, the fit contains two components: 1) a species (blue) with short decay time constant that largely constitutes emission centered at 580 nm and 2) a species (orange) with a decay time constant of 1200 ps that constitutes broad, red emission from 600 to 700 nm. When oligomer length is increased from n = 2 to n = 3, the two components are largely retained with key differences only in the peak position and decay time constants. Each component is red-shifted, most notably the longer-lived (orange) peak, which shifts 1144 cm-1 from a maximum at 618 to 665 nm.

This shift in the long-lived species can begin to explain the discrepancy between the peak maxima trends in absorption and fluorescence spectra discussed above. It has been widely reported that exciton formation in conjugated polymers tends to trigger transformation of the conjugated backbone from a benzoidal to a quinoidal form, resulting in a planarized backbone [28,29]. In fact, this change was a guiding principle in the design of **PTB7 [30,31,32]** that appears to be retained in the oligomer series. This planar conformation of the excited state potential energy minimum allows for increasing π-conjugation in the excited state that overcomes the conformational disorder inherent to the ground state and leads to the continuing red-shift with increasing n seen exclusively in the fluorescence spectra. These long-lived, red-shifted (orange in decay-associated plots) spectral features can then be attributed to the emission from the planar, quinoidal excited state. The magnitude of the red-shift of the steady state fluorescence maxima with increasing n will involve a natural red-shift of this emitting feature and the relative intensity of this quinoidal state relative to the blue emitting species. Because the overall photophysical behavior is otherwise retained, the shifting of the emission maxima of this species appears to be the primary effect of chain lengthening in the absence of aggregation.

Experiments with higher time resolution were required to identify the blue fluorescent species and provide further evidence for the identification of the quinoidal excited state. Towards this end, transient absorption (TA) and a higher time resolution streak camera experiment were conducted with specific emphasis on the n = 2 oligomer for two reasons: 1) besides peak shifting and small lifetime differences, ultrafast dynamics are similar between n = 2 and n = 3 oligomers, and 2) the n = 2 oligomer has its emission spectrum aligned with that of the corresponding polymer, and hence, is a useful model for unfolded portions in **PTB7** [20]. The parallel results for n = 3 oligomer are available in the SI (Figures S18–S22, Tables S8–S12).

**Figure 3:**
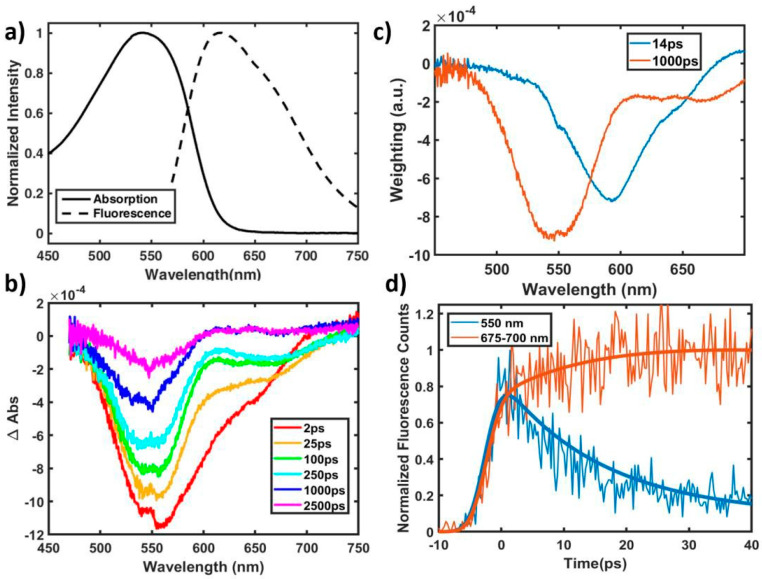
(**a**) Normalized absorption and fluorescence spectra of n = 2 oligomer. (**b**) TA spectra of n = 2 oligomer at various time delays at a pump wavelength of 550 nm. The negative signal includes contributions from both ground state bleach and stimulated emission. There is a growth in transient signal at 675 nm between 2 ps and 25 ps. (**c**) Decay-associated fitting of visible Transient Absorption data of n = 2 oligomer. The orange curve, with a strong contribution to the ground state bleach and broad stimulated emission component has a lifetime of 1 ns. The blue curve, contributing weakly to the ground state bleach and strongly to a sharp stimulated emission feature centered at 600 nm, has a lifetime of 14 ps. There is a positive feature redder than 650 nm indicative of a rise. (**d**) High time resolution streak camera traces of the n = 2 oligomer at 500 nm excitation. These traces concentrate on the edges of the fluorescence spectrum to separate contributions from the two species, at the cost of lower overall counts. Thicker lines represent the fit (details available in SI Figures S4 and S5, Table S4) with data as the thinner lines. The integrated and normalized 675–700 nm region has a growth concurrent with the 550 nm decay.

Complementary to the time-resolved fluorescence measurements, we also obtained ultrafast transient absorption spectra of the n = 2 oligomer in the visible region at 550 nm excitation. It features both ground state bleaching (GSB) from 500–600 nm and stimulated emission (SE)/excited state absorption (ESA) from 550 to 750 nm as shown in Figure 3b, mirroring their respective steady state features above in Figure 3a. There is a clear distinction in time evolution of the spectral features in the region around 600 nm with much faster decay than the rest of the spectrum, leaving behind spectrally distinct bleaching and emission peaks at later times. These data were fit globally, yielding the two-component decay-associated spectrum in Figure 3c (see SI Figure, Table S1 for fitting details).

The decay-associated fitting again reveals two characteristic spectra with two respective decay time constants, 14 and 1000 ps. The interpretation of transient absorption spectra is more complex due to the overlapping GSB, SE, and ESA spectral features. The ~1000 ps orange feature constitutes a majority of the ground state bleaching signal, overlapping nicely with the ground state absorption spectrum and identifying this pathway as the main avenue for relaxation to the ground state. It also has a lifetime and broad emission component from 600 to 700 nm similar to the red-shifted emission (Figure 2b) attributed to the planar, quinoidal excited state. Meanwhile, the blue decay component in Figure 3c largely constitutes emission at 600 nm, spectrally similar to the blue-shifted fluorescence component (Figure 2b), which can now confidently be assigned a lifetime of 14 ps. From 500 to 600 nm, this blue component has a small contribution to the ground state bleach feature, indicating some minority relaxation directly to the ground state. Evidence for the majority relaxation pathway of this species can be seen in the positive feature between 650–700 nm. Because the transient signal is negative, this positive feature indicates a growth in that region during that 14 ps time component. Evidence for this growth exists at 675 nm in Figure 1b, where the signal increases in magnitude between the presented 2 and 25 ps time cuts. Taken as a whole, it seems the 14 ps, blue-shifted species primarily decays into the 1000 ps, red-shifted species.

These observations are best understood in the context of excited state dynamics in an ensemble of conformational isomers with varying degrees of planarity. As previously discussed, the oligomer ground state allows for inhomogeneity in dihedral angle and π-conjugation between adjacent benzodithiophene (BDT) and thienothiophene (TT) units, while the quinoidal character of the excited state moves the energetic minimum towards more planar conformations. As such, the excitation pump pulse will initially excite an ensemble of oligomers with varying dihedral angles at various distances from their new, planar, excited state energetic minimum. The shorter lifetime, blue-shifted emission species are composed of those oligomers that are more severely twisted upon excitation, and their 14 ps lifetime is not a “fluorescence lifetime,” which is typically much longer for conjugated organic chromophores [33,34], but is instead their torsional relaxation time, in line with timescales seen in thiophene-based oligomers [28,29]. As evidenced by their presence in fluorescence spectra, there is some emission from these species during their relaxation period, but a majority torsionally relax into the ~1-ns, quinoidal, planar excited state. The longer-lived, red emission, then, is the primary relaxation pathway of the oligomer and is composed of both those oligomers that were relatively planar upon initial excitation and those that underwent dynamic planarization.

To directly observe this conversion from twisted to planar excited species, we carried out a second streak camera measurement with the overall time window of just ~100 ps, with ten times enhanced time resolution compared to the first set of measurements (see details in Methods section). This set of measurements enabled us to observe kinetics for the blue-shifted, twisted species while compromising the measurements for the longer component, which had a lifetime longer than the observation window. Figure 3d shows traces of the far blue and far red edges of the fluorescence spectrum of the n = 2 oligomer and their fit lines, chosen despite their comparatively low signal to noise ratio because they contain emission exclusively from the twisted and planar species, respectively. The growth of both regions begins concurrently but diverges after a few ps. While the blue emitting species reaches its maximum and begins to decay within the instrument response time, the redder species shows a secondary growth, not reaching its maximum value until the blue species has decayed and providing direct evidence for interconversion over the course of 10–20 ps. Fluorescence upconversion scans, which have much better time resolution and are available in the SI (section S1d), largely agree with kinetics observed in the streak camera and provide additional evidence of dynamic planarization.

### 2.2. In-Chain Effects on CT Character.

**Figure 4:**
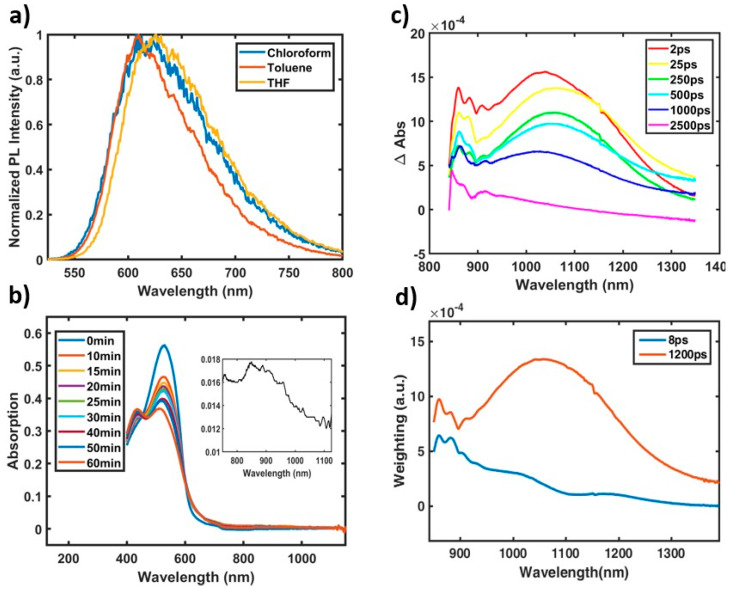
(**a**) Integrated, normalized time-resolved fluorescence from 200 ps onwards of n = 2 oligomer at 500 nm excitation in solvents of varying polarity. Starting at 200 ps eliminates contributions from the short lived, twisted species. The red edge shifts with increasing polarity. (**b**) Spectro-electrochemistry measurements of the n = 2 oligomer held at 2V oxidative potential for 1 hr. There is a blue-shift of the absorption maximum to 450 nm that is not seen in visible transient absorption. There is also a weak absorption feature that grows in the near-infrared (inset—separate scan held to allow for increased integration time). This feature does not align with either transient near-infrared feature. (**c**) Near-infrared probe Transient Absorption spectra of the n = 2 oligomer at various time delays at a pump wavelength of 550 nm. The positive signal originates from excited state absorption. Oscillatory features from 850–900 nm are artifacts of white light generation. (**d**) Decay-associated spectra of near-infrared transient absorption data. Similar to visible transient scans, there are both a short lived and longer-lived species. Details are available in the SI Table S2. Evidence of dynamic planarization.

**PTB7′**s intrinsic backbone charge transfer character has been credited with many of its unique properties implicated in its high efficiency exciton splitting and photovoltaic device performance [24,25,35]. As such, the exact nature of this charge transfer character has been of great scientific interest. However, studies have been hampered by one simple fact: **PTB7** is notoriously insoluble in all but a few chlorinated solvents [9], making it difficult to observe solvent-dependent emission shifts that reflect charge transfer characteristics [22]. The **PTB7** oligomer series, then, allows for a unique opportunity to study charge transfer character in the **PTB7** exciton for two reasons: (1) they retain solubility in a variety of solvents and (2) the geometry of their flattened, quinoidal excited state resembles the self-folded **PTB7** exciton. To obtain an accurate comparison with the polymer, fluorescence streak camera measurements were carried out with a time window extending to 1200 ps and a pump wavelength of 500 nm in solvents of varying polarity, and the contribution of the longer-lived, quinoidal excited state was isolated by integrating and normalizing intensities from 200 ps onwards, as shown in Figure 4a. The most notable shift occurs on the red edge of the fluorescence spectrum, where the polar solvents have their edge shifted ~500 cm^−1^ (20% peak height at 705 nm in toluene vs. 731 nm in chloroform/THF), indicating an increase in dipole moment of the quinoidal excited state relative to the ground state [36] and substantial charge transfer character. This indicates that **PTB7′**s polar exciton is inherent to its “push–pull” backbone structure.

### 2.3. Aggregation Effects on CT Character.

Beyond a polar exciton, a defining feature of **PTB7′**s near-infrared probe ultrafast spectra is its characteristic polymer cation peak [22,37,38], in which its “charge transfer” and “charge separated” states were detected and described as excitons that, almost instantaneously after formation, split into holes and electrons with varying extents of separation. Importantly, these spectral features were identified by comparisons to polymer cations observed in both bulk heterojunction films and those obtained via spectro-electrochemical measurements [22,38]. Because analogous charge generation has also been seen in neat conjugated homopolymers in film (but, to our knowledge, not in solution) without this “push–pull” structure [39], it is reasonable to ask if this ultrafast charge generation is a property inherent to **PTB7′**s backbone or if it is an emergent property of the self-folded polymer chain. If ultrafast charge separation occurs in the oligomers akin to that seen in the polymer, there should appear transient signal of charged oligomer, especially the cation which is known to appear in the spectrally accessible near-infrared region [38].

To obtain the spectra of the oligomer cation, spectro-electrochemical measurements were performed, as shown in Figure 4b. Two changes were observed: blue-shifting of the absorption maximum and small induced absorption at ~900 nm (Figure 4b, inset). Importantly, this blue-shifted absorption was not observed in visible TA experiments as detailed above (Figure 3b), providing evidence against the existence of charged oligomer species.

For further comparison to the ~900 nm cation peak (Figure 4b, inset), ultrafast TA experiments were conducted on the n = 2 oligomer with a near-infrared probe, as shown in Figure 4c, with decay-associated fitting in Figure 4d. There are two broad features in the TA spectrum in Figure 4c, centered blue of 875 nm and at 1100 nm. Noting that the time constants for these species are comparable to those seen in the visible region discussed earlier, these excited state absorption features are assigned to the twisted (875 nm) and planar (1100 nm) excited species. Further confirmation of the 1100 nm peak as the planar excited species is seen in its spectral red-shifting during the first ~20 ps with the dynamic planarization process, as seen clearly in Figure 4c and represented by the positive feature of the 8 ps component in the decay-associated spectrum in Figure 4d at ~1000 nm. Because neither excited state absorption signature aligns with the near-infrared spectrum obtained for the oligomer cation, the near IR probe TA experiment indicates no photoinduced formation of oligomer cation species. In fact, the ~1100 nm excited state absorption of the n = 2 oligomer observed in the near-infrared is consistent with linear response calculations of the excited singlet absorption of a short oligomer of **PTB7**, [40] indicating that the planar, quinoidal species is a singlet excited state and that the progression from charge transfer character to charge separation is modulated by the self-folded polymer structure. Importantly, the appearance of charge separation only in the self-folded polymer does not assert that exciton splitting necessarily occurs only across multiple chains, as the enhanced π-conjugation in the polymer could allow further modulation of the charge transfer character of the backbone beyond that seen in even the n = 2 and n = 3 oligomers.

The identity and properties of the ~1100 nm excited state absorption signature in neat **PTB7** is still an area of active research, due to overlapping spectral signatures of the polymer triplet absorption [41] with our assignment of the polymer cation [22,38] and recent work suggesting the ultrafast creation of symmetry conserving triplet pairs in **PTB7** film [42]. While the presence of triplets at longer times in **PTB7** is uncontroversial [41], we contend that the species produced in the ultrafast regime is a charge separated state despite the energetics of exciton splitting without electron acceptor at first seems energetically unfavorable: **PTB7** has been estimated to have thermally inaccessible exciton binding energies of ~0.4 eV [42] or as low as ~0.2 eV [43] at low torsional angles. Nevertheless, measurements with externally applied fields did not observe the step-like transition predicted by a simple Onsager–Braun model with these kinds of splitting energies [44] at the applied field value that overcomes the exciton binding energy. Instead, it was observed that exciton splitting shown large dependencies on disorder and a gradual onset with respect to the applied field at energies lower than those predicted by an Onsager–Braun model. This suggests an inherent sensitivity to the polymer local environment and site energies [44], as small fractions of polymer meet the energetic requirements for exciton splitting before the bulk film. In fact, this may suggest a mechanism by which aggregation modulates the CT character inherent to the **PTB7** backbone discussed in this manuscript. Variations in π-conjugation length in **PTB7** chains have shown very large energetic shifts via the oligomer series, with over 0.37 eV bandgap shifts between oligomer and polymer [20]. Assuming symmetrical movement of HOMO and LUMO levels, this kind of shift could lead to relative movement of ~0.19 eV of polymer and oligomer frontier orbitals. The presence of solution aggregates provides both the energetic shifts and disorder in site energies necessary for exciton splitting. Nevertheless, these transient species, and any long-lived spectroscopic species, are not present in the oligomers, as shown in this work. Regardless of the mechanism, the modulation of energetics via aggregation and local disorder is necessary for whichever process dominates.

## 3. Materials and Methods

### 3.1. Materials

PTB7 oligomers were designed with alternating benzodithiophene (in chain donor, D) and fluorinated thienothiophene (in chain acceptor, A) units in a (D-A)_n_-D arrangement, where n = 1–3. The oligomers will be referred to as (BDT-TT)_n_BDT (benzodithiophene-thienothiophene), with the n specified or, more broadly, as the PTB7 oligomer series. Oligomers were synthesized using a Stille coupling reaction between stannated benzodithiophene and brominated thienothiophene units. Due to the presence of only two singlet peaks in the aromatic region of the ^1^HNMR spectra and one singlet in the 19FNMR spectra, it is concluded that there is only one isomer present in oligomer samples. For a more detailed description, see the previous report [20]. Chloroform and chlorobenzene were purchased from Sigma-Aldrich (anhydrous > 99%). For the comparison studies, PTB7 was purchased from 1-Material (http://www.1-material.com/).

### 3.2. Steady State Spectroscopy

UV-Visible spectra were taken on a Shimazdu UV-3600 Spectrophotometer. PTB7 samples were prepared at a concentration of 0.1 mg/mL in chloroform for an absorption maximum of ~0.7 in a 2 mm path length quartz cuvette. Oligomer samples were prepared in chloroform at a concentration of 0.12 mg/mL for an absorption maximum of 0.5 in a 2 mm path length quartz cuvette. Fluorescence spectra were taken on a Fluorolog-3 Spectrofluorometer at a 90 degree detection angle. Samples were prepared in chloroform and analyzed in a 1 cm fluorescence cuvette.

### 3.3. Spectro-electrochemistry

Spectro-electrochemical measurements were undertaken to acquire the absorption spectrum of oxidized PTB7 oligomer to compare with transient spectra. Samples were prepared at a concentration of 0.8 mg/4 mL in dichloromethane with 0.1 M electrolyte. Using a VersaSTAT 4 potentiostat (Princeton Applied Research, Oak Ridge, TN, USA), a 2V oxidative potential was held over time while monitoring the absorption using the Shimazdu UV-3600 Spectrophotometer discussed above. Subsequently, the potential was released, and absorption was monitored to make sure no irreversible damage came to the sample (SI Section S1b). The reference electrode was Ag/AgCl in KCl.

### 3.4. Ultrafast Transient Absorption Spectroscopy

Transient Absorption (TA) measurements were performed on solutions of PTB7 and PTB7 oligomers in chloroform, unless noted otherwise, using an ultrafast laser system (Coherent, Santa Clara, CA, USA), as described previously [45]. Briefly, the system includes a Ti:sapphire oscillator (Mira) pumped by a diode laser (Verdi-5) and a regenerative amplifier (RegA 9050) pumped by a diode laser (Verdi-10) with a separate compressor/stretcher and optical parametric amplifier (Coherent). The amplified output of RegA (10 uJ/pulse at 100 kHz repetition rate) was compressed to ~50 fs and divided with an 80/20 beamsplitter. The lower power portion of the fundamental beam was tightly focused on a 3 mm thick yttrium aluminum garnet (YAG) plate to generate visible and near-IR probe, adjusting focusing to switch between probe regions (400–800 nm vs. 850–1400 nm). The higher power portion of the fundamental beam was used to pump the Optical Parametric Amplifier (Coherent, OPA) to generate the excitation pulse from 500–650 nm.

The signal was spectrally dispersed with a monochromator grating and detected using either a Pixis 100 CCD camera array for the visible or a PyLoN-IR InGAs camera (Princeton Instruments) for the near-infrared. A custom Labview (National Instruments, Roscoe, IL, USA) program was used to interface with the native camera software (LightField, Princeton Instruments, Trenton, NJ, USA), control the pump pulse delay stage, and manage timing. The pump beam was chopped at 500 Hz, allowing 200 pulses per cycle. In total, 10,000 frames were collected, averaged, and subtracted for each data point. Scans consisted of 256 time steps and extended to 3 ns. TA spectra shown in the paper consist of a minimum of 3 averaged scans. The pump and probe beams were set to the magic angle relative to each other with a waveplate.

Samples were prepared to have OD = 0.4–0.7 at their peak in visible absorption spectrum in a 2 mm path length quartz cuvette. Samples were purged with nitrogen, sealed, and stirred during transient absorption measurements. In polymer samples, pump energies were kept below 4 nJ/pulse with a 100 μm spot size to avoid second and higher order processes. Oligomer samples were allowed 10 nJ/pulse to maximize signal without degrading the sample.

### 3.5. Streak Camera Fluorescence Measurements

Samples were prepared in chloroform as above. The samples were photoexcited using a 500 Hz, 35 fs Ti:sapphire amplifier. The 800 nm output of the laser pumped an OPA to produce 500 nm light which photoexcited the sample. The photoluminescence photons were directed through a 550 nm long pass filter and into a 150 mm spectrograph and single-photon-sensitive streak camera. Detector regions were binned vertically or horizontally to produce time-resolved spectra or spectrally-resolved dynamics. Longer observation windows necessarily yield reduced temporal resolution and vice versa, so dynamics which appear well resolved in a short total time scan can appear instrument limited in longer observation windows [46]. As such, full time scans had a total time of 2091.6 ps, leading to a ~20 ps time resolution.

### 3.6. Data Analyses.

TA spectroscopy and time-resolved emission from streak camera data were treated using software developed in MATLAB [47]. Before fitting, Transient Absorption data were corrected for background and chirp. Then, the data were fit globally to a sum of two exponential decay functions in all cases, assuming a uniform instrument response and time zero across the probe wavelength range, with the following equation:

*I*(*λ*, *t*) = Σ *A*(*λ*, 0)(*e*^*−t/τn*^ ∗ *IRF*(*t*)) (1)

where n = 2. Time constants are fit globally, while the amplitudes of the exponential functions vary with the probe wavelength. The dataset was then deconvoluted with the resultant amplitudes to reconstruct the decay-associated spectra. Figures plotted in the text, then, show the wavelength-dependent basis spectra (A(*λ*, 0)) at time zero that evolve monoexponentially with the time constant listed in the legend [48,49]. IRF details are available in the SI Section S4.

## 4. Conclusions

A series of oligomers with the same alternating electron donor–acceptor block sequence as that of **PTB7** was studied using ultrafast measurements to isolate and determine the effects of chain lengthening and aggregation on photophysics and kinetics. The two longer oligomers, with chain length n = 2 and n = 3, were shown to be useful analogues of unfolded segments of **PTB7** and retained similar absorption spectra and consecutively red-shifted emission. This emission shift was explained by dynamic backbone flattening in the excited state with quinoidal character that allowed for π- conjugation greater than that seen in the more conformationally disordered ground state. This planar excited species with ~1 ns lifetime was noted to be similar to the exciton of **PTB7** in the absence of interchain interactions, whose self-folding enforces a similar planar geometry.

To investigate whether **PTB7′**s charge generation is related to its unique backbone structure or aggregation state, the emission spectrum of the planar, quinoidal excited state in the n = 2 oligomer was isolated from more twisted conformers in solvents of varying polarity. The solvent polarity-dependent peak shifting confirms that the primary effect of the “push–pull” motif of the backbone is the creation of substantial charge transfer character in the exciton. Further to this, the lack of spectral evidence for long-lived, charged oligomer suggests that interchain interactions via π–π stacking in **PTB7** are required for ultrafast generation of the charge separated state via a mechanism similar to neat charge photogeneration in polymer films [44]. These results could provide important correlations between the structural dynamics in conformationally diverse **PTB7** and its exciton dynamics, and probe the evolution of charge transfer character during a change in aggregation state, suggesting that fundamental electronic structure in both free chains and aggregates must be understood and controlled to further improve photovoltaic performance.

## Supplementary Materials

The following are available online. The Support Information is available online. 1. Supplemental Spectra of N = 2 oligomer(S2-S11): a. Fitting Details for N = 2 oligomer; b. Spectroelectrochemistry; c. Solvent Dependent Behavior; d. Fluorescence Upconversion; 2. PTB7 Oligomer Length Dependent Measurements (S12-S18): a. Transient Data and Fitting for Oligomer Series, b. Transient Solvent and Pump Dependence for n = 3 Oligomer; 3. PTB7 Transient Spectra (S19–S22): a. Fitting Details for PTB7; b. PTB7 Power Dependence, c. PTB7 Near Infrared Probe Transient Absorption; 4. Global Analysis Details (S23).
