# Supplementary material for: Effects of Intra- and Interchain Interactions on Exciton Dynamics of PTB7 Revealed by Model Oligomers
Source: Molecules. 2020 May 23;25(10):2441. doi: 10.3390/molecules25102441 (PMC7287679; doi:10.3390/molecules25102441)
Supplement: Supplementary file 1 [file molecules-25-02441-s001.pdf]

SUPPORTING INFORMATION

Aggregation Induced Progression of Charge Transfer Character and Ultrafast Dynamics in Organic Semiconducting Polymer PTB7 Revealed by Oligomer Models

Thomas J. Fauvell[a,b,c], Zhengxu Cai[d], Matthew S. Kirschner[a,c], Waleed Helweh[a], Pyosang Kim[a,c], Tianyue Zheng[d], Richard D. Schaller[a,c], Luping Yu[b,d], and Lin X. Chen*[a,b,c]*

[a]Department of Chemistry, Northwestern University, 2145 Sheridan Road, Evanston, Illinois 60208, United States

[b]Argonne-Northwestern Solar Energy Research (ANSER) Center, Northwestern University, 2145 Sheridan Road, Evanston, Illinois 60208, United States

[c]Chemical Sciences and Engineering Division, Argonne National Laboratory, 9700 South Cass Avenue, Argonne, Illinois 60439, United States

[d]Department of Chemistry and James Frank Institute, The University of Chicago, 929 East 57th Street, Chicago, Illinois 60637, United States

<u>Table of Contents</u>

1. Supplemental Spectra of **N=2 oligomer**(1-11)
    a. Fitting Details for **N=2 oligomer**(1-5)
    b. Spectroelectrochemistry (6)
    c. Solvent Dependent Behavior (6-7)
    d. Fluorescence Upconversion (7-11)
2. **PTB7** Oligomer Length Dependent Measurements (11-16)
    a. Transient Data and Fitting for Oligomer Series (11-15)
    b. Transient Solvent and Pump Dependence for n=3 Oligomer (15-16)
3. **PTB7** Transient Spectra (17-20)
    a. Fitting Details for **PTB7** (18-19)
    b. **PTB7** Power Dependence (18)
    c. **PTB7** Near Infrared Probe Transient Absorption (19-20)
4. Global Analysis Details (20)

Supplemental Spectra of N=2 Oligomer

Fitting Details for N=2 Oligomer

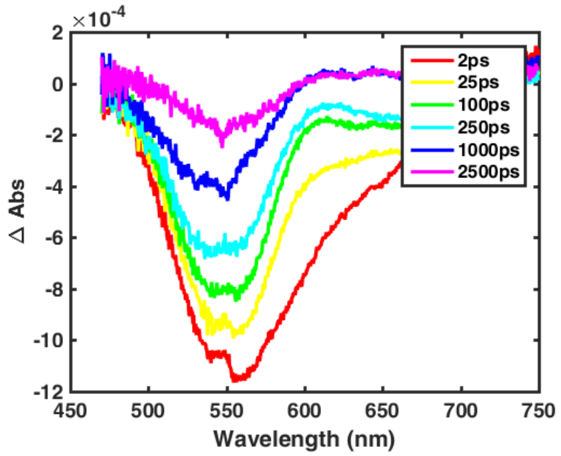

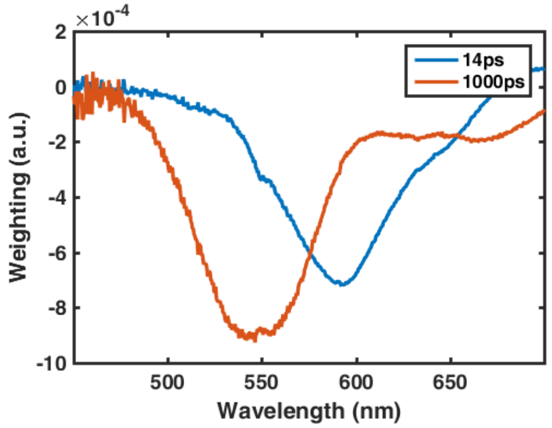

Fig S1 - (Top) Visible Probe Transient Absorption spectra of **N=2 Oligomer** in chloroform at various time delays at a pump wavelength of 550nm. (Bottom) Decay Associated fitting of transient data. Reproduced from the main text

Fit Parameter	Value
MSE	7.1786e-09
tau_1	13.9860 +- 7.8003e-01
tau_2	977.5028 +- 5.0148e+01
t0	-0.0373 +- 4.7851e-03
irf	0.1172 +- 1.5220e-02

Table S1 - Parameters for above fit. Mean Squared Error (MSE) measures the averages of the square of the errors between the fit and the data. A lower number is better, and its magnitude should be compared to the size of the data.

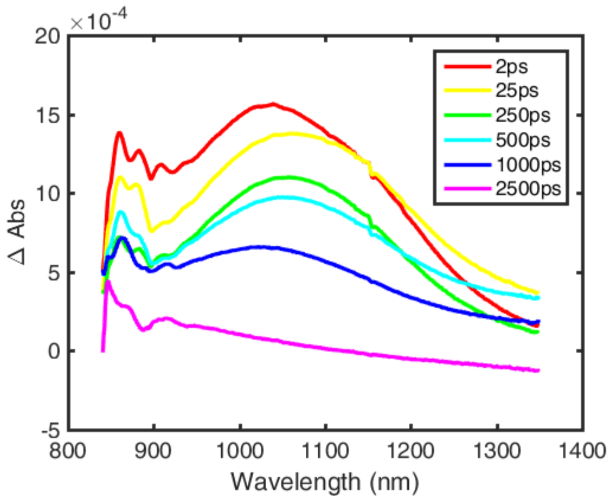

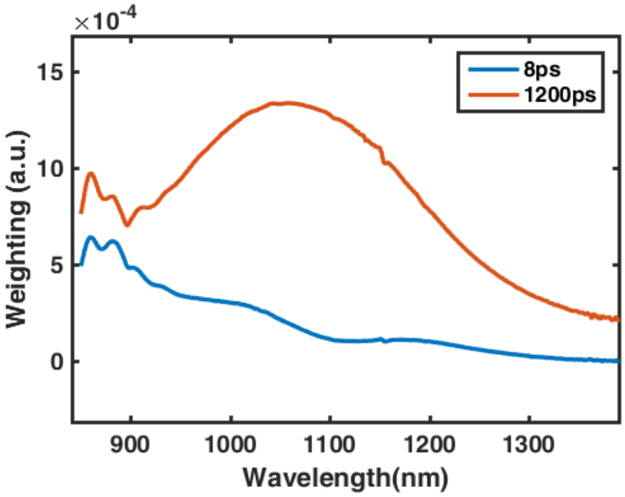

Fig S2 - (Top) Near Infrared Probe Transient Absorption spectra of **N=2 Oligomer** in chloroform at various time delays at a pump wavelength of 550nm. The identity of this species was inferred by comparing to spectroelectrochemistry (SI Sec 1b and main text Fig 3c). (Bottom) Decay Associated fitting of transient data. Reproduced from the main text.

**Fit Parameter**	**Value**
MSE	5.0225e-09
tau_1	8.2108 +- 6.3919e-01
tau_2	1291.5580 +- 3.0511e+01
t0	0.0027 +- 2.4810e-03

Table S2 - Parameters for above fit

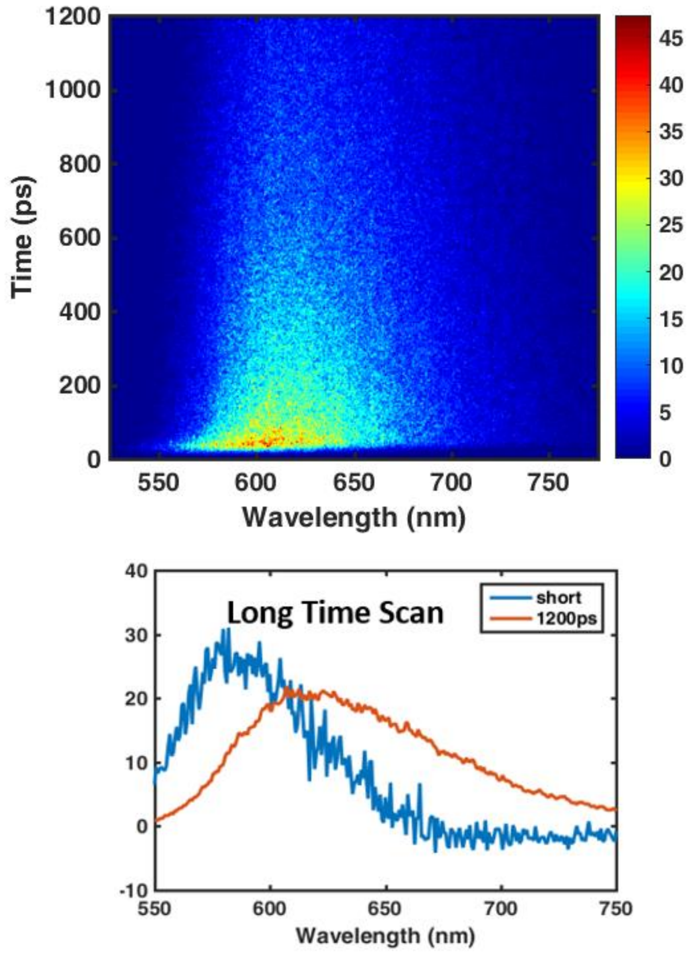

Fig S3 – (Top) Full Time Streak Camera Data. (Bottom) Decay associated fit. (Reprinted from Figure 2 in main text)

Fit Parameter	Value
MSE	1.1414e-04
tau_1	39.0762 +- 1.0374e+00
tau_2	1235.6580 +- 3.5344e+01
t0	71.9339 +- 2.7741e-01
IRF	16.8859 +- 8.0514e-01

Table S3 - Parameters for streak camera fit shown in main text Figure 2b. The 39 ps component fit here is too short to be deemed reliable on a streak camera measurement with this time window, regardless of the confidence of the fit. It should be combined with the shorter window experiment below.

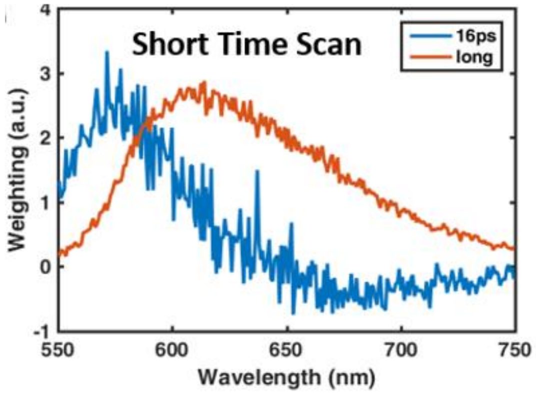

Figure S4 – Decay Associated Fitting for Short Time Window Scan. This is the short time (high time resolution) scan equivalent of Figure 2b from the main text. The relative positions and sizes are similar. Fitting parameters are below in Table S4

Fit Parameter	Value
MSE	2.2975e-04
tau_1	16.1806 +- 7.2644e-01
tau_2	473.2168 +- 1.9766e+02
t0	15.6896 +- 1.1282e-01
IRF	2.3367 +- 3.4383e-01

Table S4 - Parameters for above fit. The long component here is too long to be deemed reliable on a streak camera measurement with this time window. It should be combined with the longer experiment above.

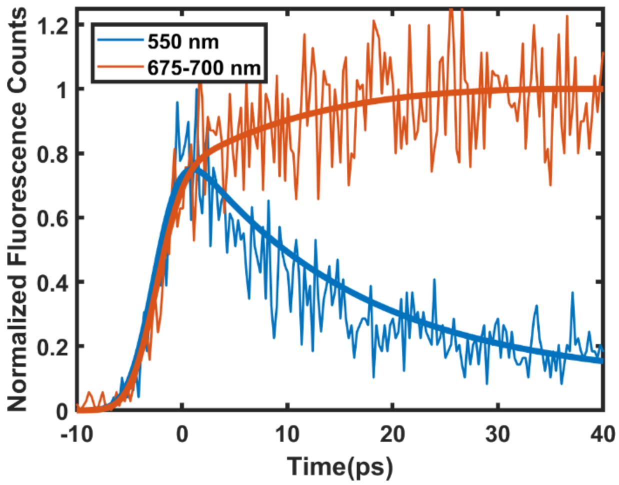

Figure S5-Reproduced from Figure 2c. Thin lines represent data while thick lines represent fits. Fits are of the form c1*(IRF*exp(-t/tau1)) +c2*(IRF*exp(-t/tau2)). The values for tau1, tau2, and IRF were taken from the fit in TableS4. The values for c1 and c2 were taken from the height of the associated curve in figure S5 for the 550 nm curve, and summed intensities of every point for both curve for the 675-700 nm curve. Both data and fit curves were scaled to appear in the same figure. Note: t0 in this figure is shifted relative to that in the fit for clarity.

Spectroelectrochemistry

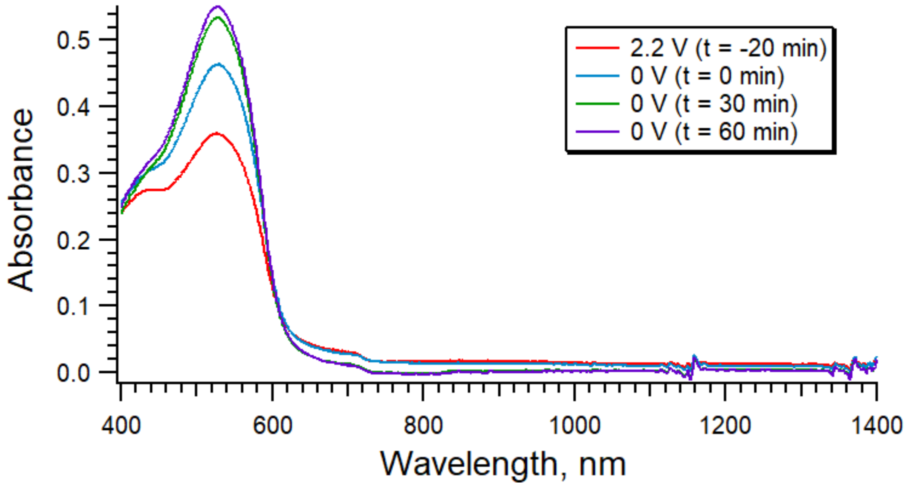

Fig S6 - Spectroelectrochemistry of **N=2 Oligomer** left after electrochemistry experiment. N=2 Oligomer recovers to its initial state, showing no irreversible product formation.

Solvent Dependent Behavior

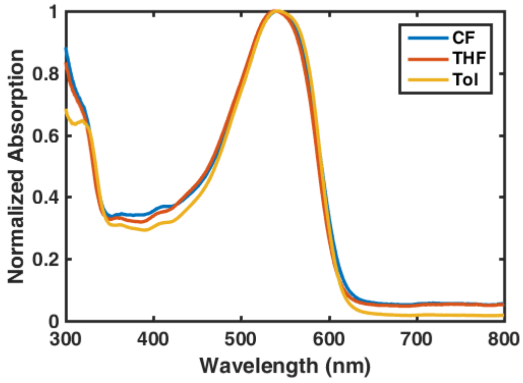

Fig S7 - Normalized absorption spectra of **N=2 Oligomer** in solvents of varying polarity. There is no appreciable peak shift.

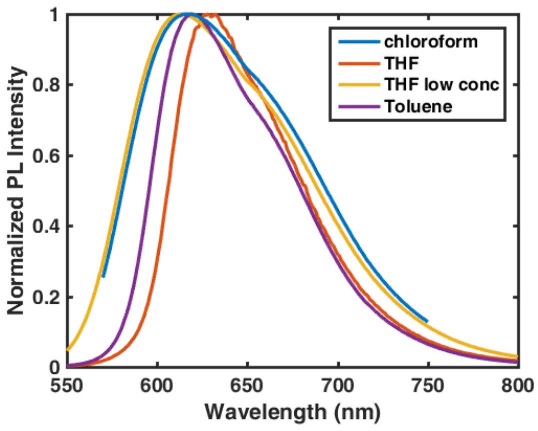

Fig S8 - Normalized emission spectra of **N=2 Oligomer** in solvents of varying polarity. There are small but definite shifts. This could be due to differing concentration dependence of aggregates in different solvents or polar character in the excited state.

High Time Resolution Streak Camera Measurements

To determine the rise components of components in the solvent dependent scans in the main text, shorter total time and, hence, higher time resolution streak camera measurements were undertaken on the **N=2 Oligomer**, with identical sample preparation. These solvent dependent traces show faster rise with higher solvent polarity, indicating some contribution of polarity to the fluorescence dynamics and can be compared with Figure 2 in the main text which was carried out in chloroform. Overall behavior is similar to that presented in the main text with some shifting conversion lifetimes, again providing evidence for a polar excited state.

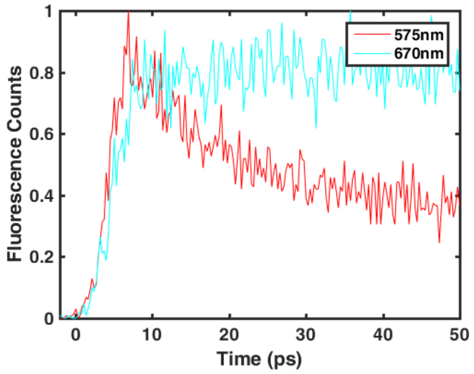

Fig S9 - **N=2 Oligomer** in THF

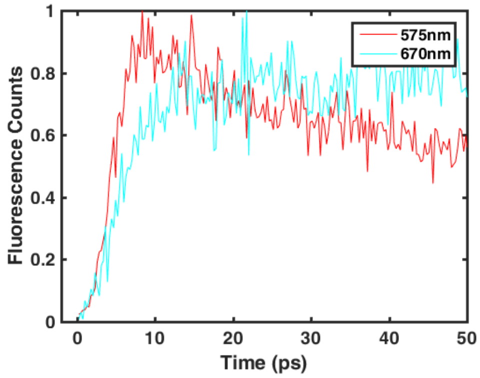

Fig S10 - **N=2 Oligomer** in Toluene

Fluorescence Upconversion

Fluorescence Upconversion experiments were carried out to utilize the enhanced time resolution of the experiment to more accurately determine time constants for the fluorescent species seen in

N=2 Oligomer. In general, the time constants garnered from this experiment line up with those seen in the main text. It was thought that these experiments could be used to accurately measure the rise of the red fluorescent species, but upconverted signal for wavelengths red of 650nm could not be distinguished from the SHG of the gate pulse. As such, while there is a noticeable trend with slower rise times towards the red edge of the spectrum, which would be consistent with the explanation shown in the main text, there is no definitive proof of the ~10ps rise, as was seen in the streak camera measurements in the main text and above.

Fluorescence Upconversion Methods

Fluorescence Upconversion measurements were performed on solutions of **N=2 Oligomer** in chloroform, prepared as detailed in the main text, using the same ultrafast laser system as in the main text for TA experiments. Instead of sending the laser light to create the white light, the lower power portion was sent directly to the experiment. Both portions (fundamental and tunable OPA output) of the laser were sent in a Halcyone (Ultrafast Systems) commercial time-resolved fluorescence system. The remaining portion of the 800 nm fundamental beam was sent to a computer-controlled delay stage to function as the "gate." The portion from the OPA was used to excite the sample and was rotated with a waveplate to the magic angle relative to the gate. Pulse energies were kept under 10 nJ/pulse. The fluorescence emitted from the sample was collected and upconverted in a nonlinear BBO crystal by mixing with the variably delayed residual 800 nm beam. The upconverted signal was collected by a monochromator and detected by a photomultiplier tube.

Representative Fluorescence Upconversion Traces with Different Time Windows

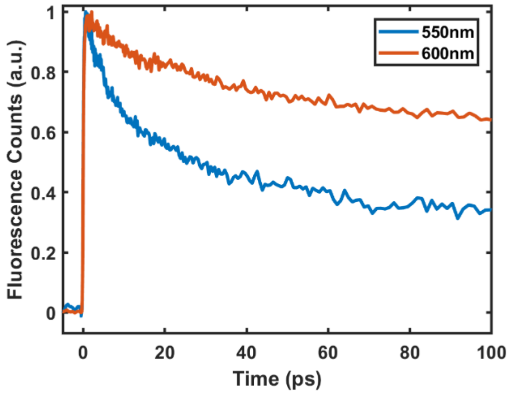

Fig S11 - Upconversion traces at 550 and 600nm, short times.

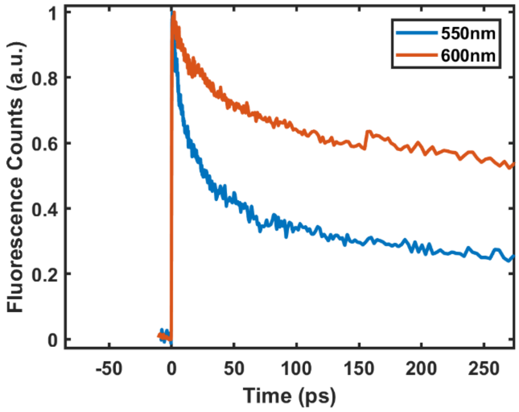

Fig S12 - Upconversion traces at 550 and 600nm, intermediate times.

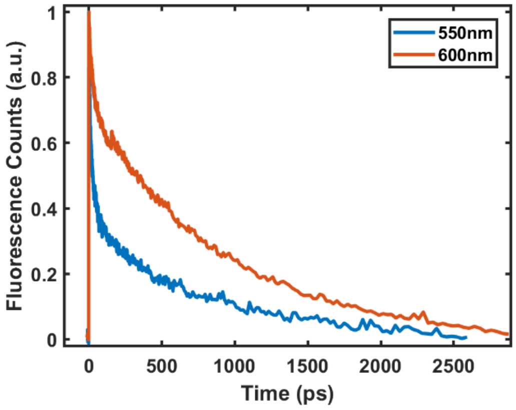

Fig S13 - Upconversion traces at 550 and 600nm, long times.

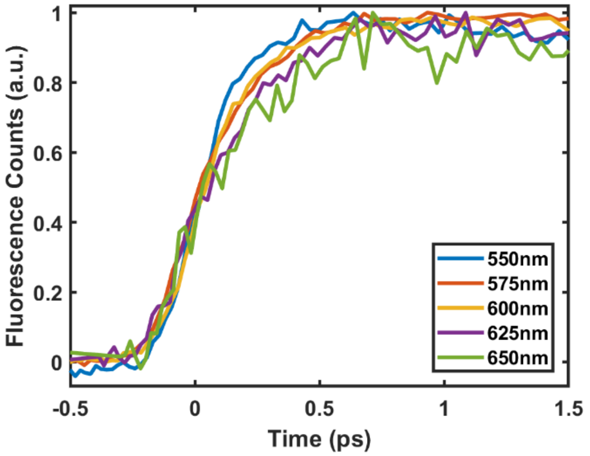

Fig S14 - Rise comparison by wavelength. Redder wavelengths show a slower rise, supporting but not confirming the dynamic flattening argument made in the main text.

Fitting

Probe (nm)	Time Zero /ps (error)	IRF (error)	Weight 1 (error)	Tau 1 (error)	Weight 2 (error)	Tau 2 (error)	MSE
550nm	0.0211 (0.0027)	0.1390 (0.0038)	0.5480 (0.0038)	15.23 (0.2769)	0.3997 (0.0031)	668.0 (12.54)	4.782 x 10^{-4}
575nm	0.0438 (0.0024)	0.1992 (0.0033)	0.3975 (0.0027)	19.15 (0.33)	0.5801 (0.0022)	779.74 (7.1)	2.329 x 10^{-4}
600nm	0.0491 (0.0020)	0.1714 (0.0027)	0.2512 (0.0025)	26.17 (0.66)	0.7148 (0.0022)	910.6 (6.5)	1.974 x 10^{-4}
625nm	0.0589 (0.0062)	0.2173 (0.0085)	0.3547 (0.0067)	17.92 (0.89)	0.5872 (0.0054)	875.8 (20.4)	1.5 x 10^{-3}

Table S5 - Fit parameters for Fluorescence Upconversion of **N=2 Oligomer** at different fluorescence wavelengths. All times are in picoseconds. Data is normalized so values from traces vary from 0-1. Data was fit to a sum of 2 exponential decays convoluted with a Gaussian IRF with width that was allowed to vary in the fitting but was expected to over 100fs (0.1+ ps). Mean Squared Error (MSE) measures the averages of the square of the errors between the fit and the data. A lower number is better, and its magnitude should be compared to the size of the data, which has a maximum defined as 1 for this data set.

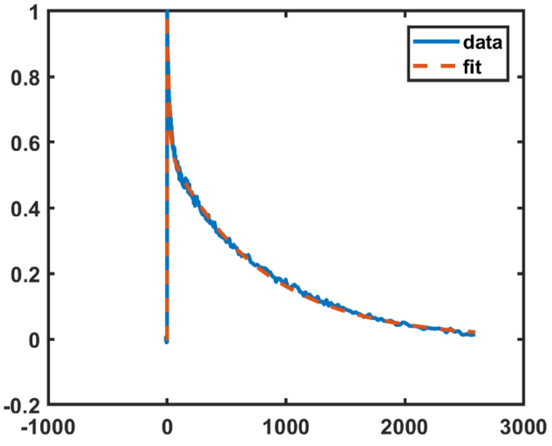

Fig S15 - Representative fitting plot. 575nm

Length Dependent Oligomer Dynamics

Transient Data and Fitting for Oligomer Series

All spectra are taken and fit using the methods and laser systems used for **N=2 Oligomer** in the main text and SI. The n=1 oligomer did not show appreciable signal in the infrared region.

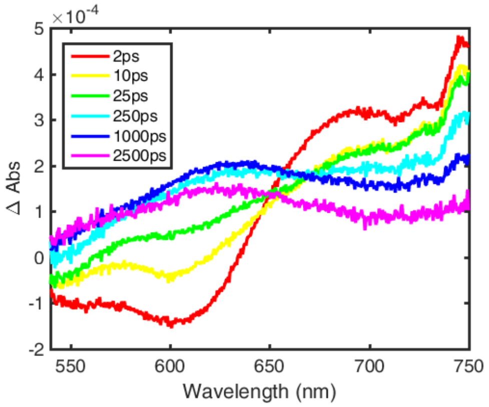

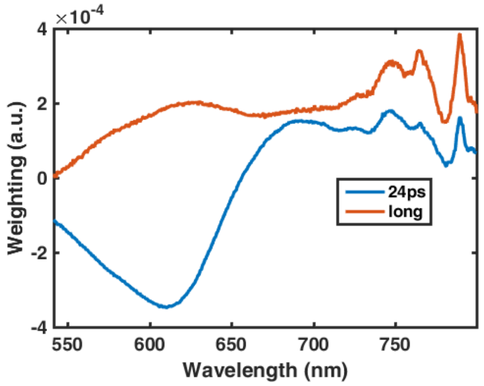

Fig S16 - Visible Transient absorption scans for **n=1 oligomer** in chloroform and decay associated fitting. The excited state absorption seen in the near infrared in other oligomers crowds the spectra.

Fit Parameter	Value
MSE	7.7856e-10
tau_1	24.4060 +- 8.8778e-01
tau_2	4191.6352 +- 2.1953e+02
t0	-0.4604 +- 4.6387e-03
irf	0.1409 +- 1.4998e-02

Table S6 - Parameters for above fit. The long-lived component is not seen in other oligomers or in Streak Camera measurements. Notably, sample was very damaged after these scans, suggesting possible photoproduct and questioning the credibility of the lifetimes.

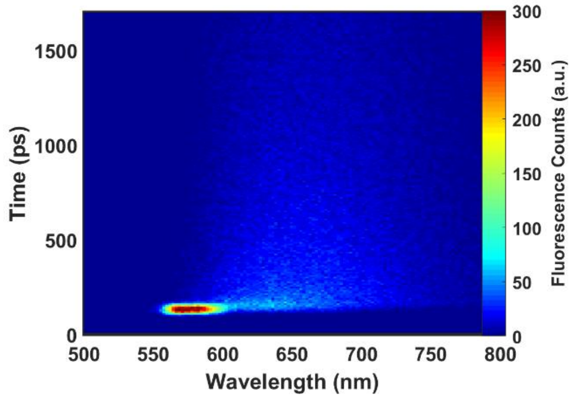

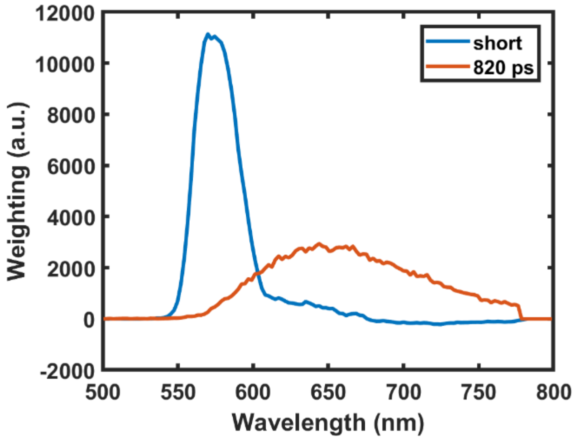

Fig S17 – (Top) Streak Camera measurements for **n=1 oligomer** in chloroform with 500 nm excitation. There is high intensity in the blue shifted peak and low intensity from the red shifted peak. This may indicate a lower quantm yield of PL in the quionoidal, planar excited species. (Bottom) Decay associated fit. The orange feature is scaled up by 100 to be shown on the same plot. The shape is reminiscent of that seen in **N=2 Oligomer**, with a fast, blue shifted component that has a negative (growth) feature overlapping with the red shifted, long-lived fluorescence.

Fit Parameter	Value
MSE	8.8284e-05
tau_1	3.9013 +- 1.9923e+00
tau_2	827.4318 +- 1.7926e+01
t0	185.8701 +- 6.3766e-01
irf	29.4967 +- 8.6176e-01

Table S7 - Parameters for above fit. In contrast to the visible transient absorption data, these data align with the other oligomers discussed in the main text. Time constants and peak positions are similar, but not within experimental error of the other oligomers.

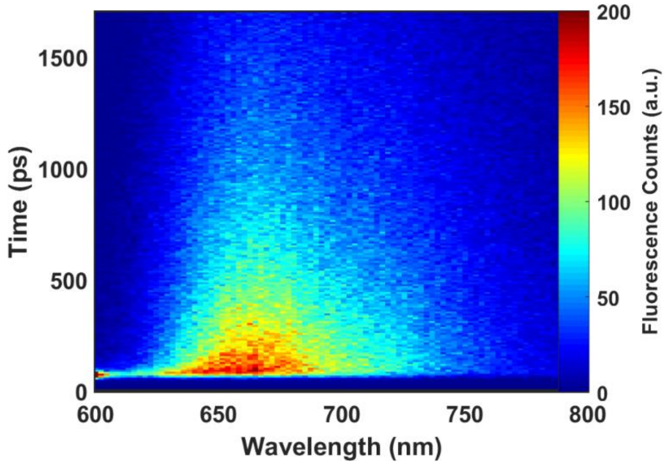

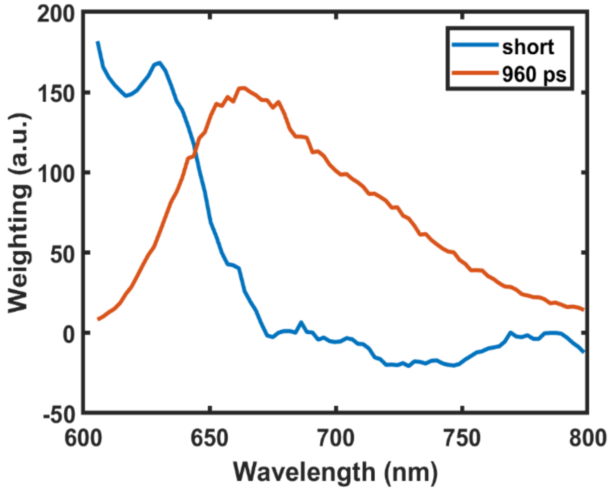

Fig S18 (Top) Streak Camera data for **n=3 oligomer** exicted at 550nm in chloroform. (Bottom) Decay Associated spectra of above scan. Both reproduced from main text

Fit Parameter	Value
MSE	5.9695e-05
tau_1	19.2158 +- 5.3911e-01
tau_2	962.2749 +- 1.9375e+01

t0	34.5183 +- 2.1596e-01
irf	20.7507 +- 5.2225e-01

Table S8 - Parameters for above fit.

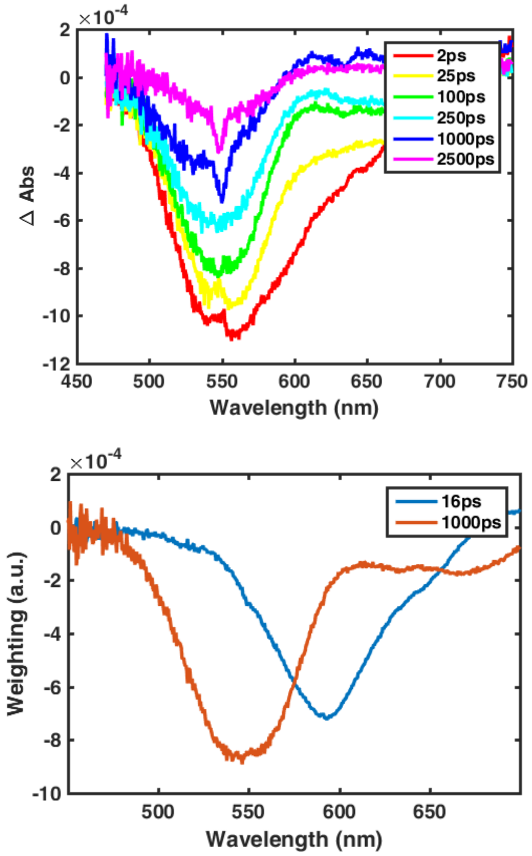

Fig S19 - (Top) Visible Probe Transient Absorption spectra of **n=3 oligomer** in chloroform at various time delays at a pump wavelength of 550nm. (Bottom) Decay Associated fitting of transient data. Similar behavior and lifetime to **n=2 oligomer**.

**Fit Parameter**	**Value**
MSE	1.0557e-08
tau_1	16.5503 +- 1.1470e+00
tau_2	1035.3914 +- 6.7832e+01
t0	-0.0426 +- 6.3887e-03
irf	0.1278 +- 2.0481e-02

Table S9 - Parameters for above fit

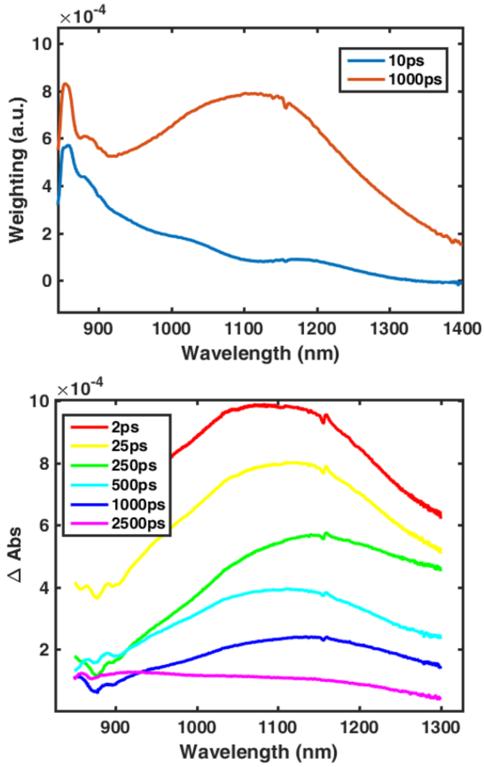

Fig S20 - (Bottom) Near Infrared Probe Transient Absorption spectra of **n=3 oligomer** in chloroform at various time delays at a pump wavelength of 540nm. (Top) Decay Associated fitting of nIR Transient data. The peak is red shifted relative to **n=2 oligomer**.

Fit Parameter	Value
MSE	8.8167e-10
tau_1	10.4141 +- 4.7473e-01
tau_2	967.2411 +- 1.5048e+01
t0	0.0139 +- 1.7199e-03

Table S10 - Parameters for above fit. The behavior is similar to the n**=2 Oligomer**.

Transient Solvent and Pump Dependence for n=3 Oligomer

To investigate the nature of the excited state, solvent and pump dependent experiments were carried out on the **n=3 oligomer**. The differences between chloroform at 540nm pump (previous section) and 610nm pump (below) were within fitting error, but there was a slight red shift and time constant change moving to a less polar solvent, indicating some CT character in the excited state. However, as discussed in the main text, this is likely not an oxidized species.

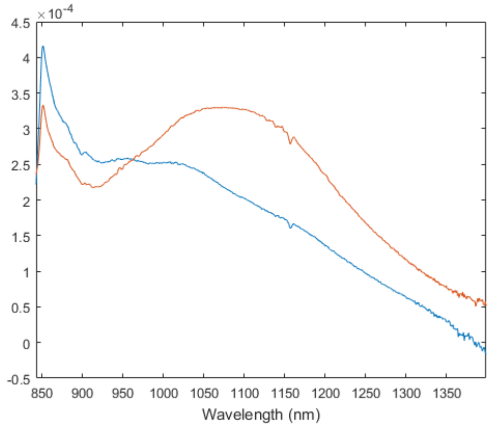

Fig S21 - Near Infrared Probe Transient Absorption spectra of **n=3 oligomer** in chloroform at various time delays at a pump wavelength of 610nm. Tau 1 is blue and tau2 is orange.

Fit Parameter	Value
MSE	7.5518e-10
tau_1	15.3878 +- 7.1906e-01
tau_2	935.2200 +- 4.8948e+01
t0	0.0137 +- 2.7949e-03

Table S11 - Parameters for above fit

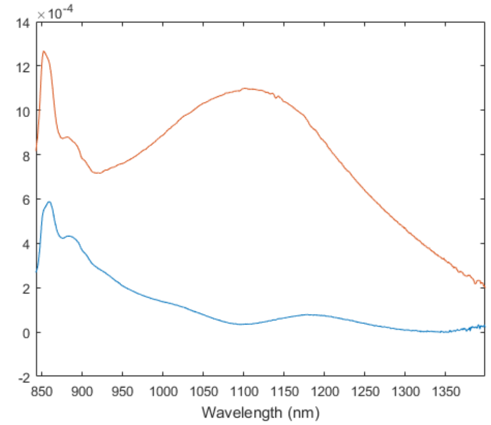

Fig S22 - Near Infrared Probe Transient Absorption spectra of **n=3 oligomer** in toluene at various time delays at a pump wavelength of 610nm. Tau 1 is blue and tau2 is orange.

Fit Parameter	Value
MSE	1.3554e-09
tau_1	7.2281 +- 4.3894e-01
tau_2	1026.1893 +- 1.3674e+01
t0	0.0091 +- 1.6961e-03

Table S12 - Parameters for above fit

PTB7 Transient Spectra

Fitting Details for PTB7

PTB7 was fit using the global, decay associated fitting discussed in the main text. Because there was not any shifting, the decay associated spectra were not provided in the main text.

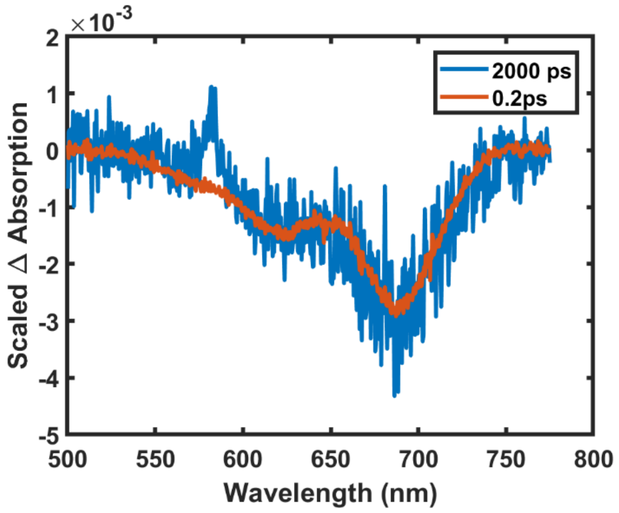

Fig S23 - Superimposed early and late time cuts of **PTB7** scans mentioned in the text. The 2000ps scan has been multiplied by 7 for comparison. Both roughly overlap, indicating no spectral shifting over the time window of the experiment, unlike the oligomers. The 2000ps time cut resembles the long time component from the decay associated fit.

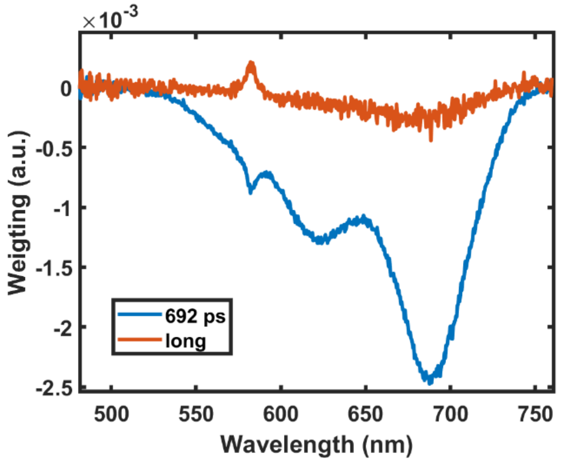

Fig S24 - Decay associated fits for **PTB7** transient absorption scans referenced in the main text (chloroform, visible probe, 580nm pump, pump power below 4nJ/pulse). Both share roughly the same shape, except the sharp feature around 580nm, which is due to randomness in the pump scatter. This was excluded from the main text because the two components are so similar, as the spectra decays homogenously.

Fit Parameter	Value
MSE	9.1522e-09
tau_1	692.1404 +- 3.2933
tau_2	Long
t0	-0.0111 +- 6.3671e-04
irf	0.2227 +- 2.0916e-03

Table S13 - Fitting Parameters for above fit. MSE is 6 orders of magnitude lower than signal intensities.

PTB7 Power Dependence

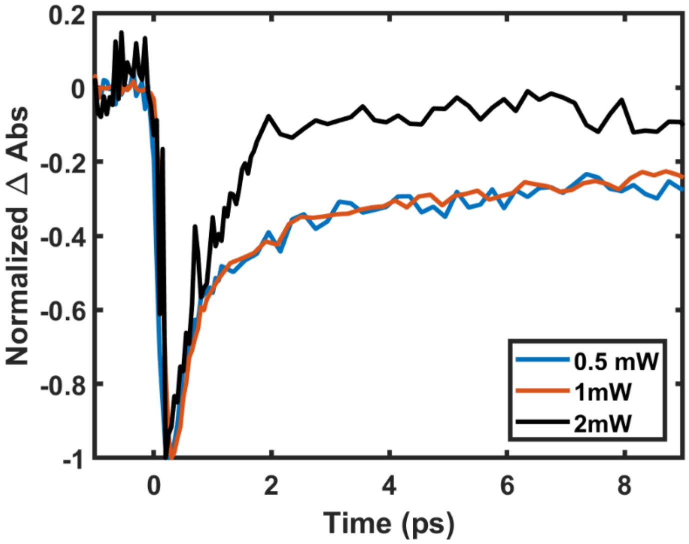

Fig S25 - Scans were carried out with the experimental setup in the text. Pump powers, as written, correspond to 5, 10, and 20 nJ/pulse, respectively. Probe wavelength is 650nm. All listed pump powers are higher than those used for analysis. These samples were unstirred, unlike those in the main text, which contributes to the fast decay seen. Past 10nJ/pulse, a change in the fast time component is evident, with high excitation energy leading to this component comprising almost all the signal. As such, this fast component can be assigned to exciton-exciton annihilation, and care should be taken to remain at excitation energies where this component is minimized. Scans presented in the main text used even lower power than is shown here to completely remove this feature. The high power scan was the average of 2 scans instead of the 4 for the others, explaining the higher noise.

PTB7 Near Infrared Probe Transient Absorption

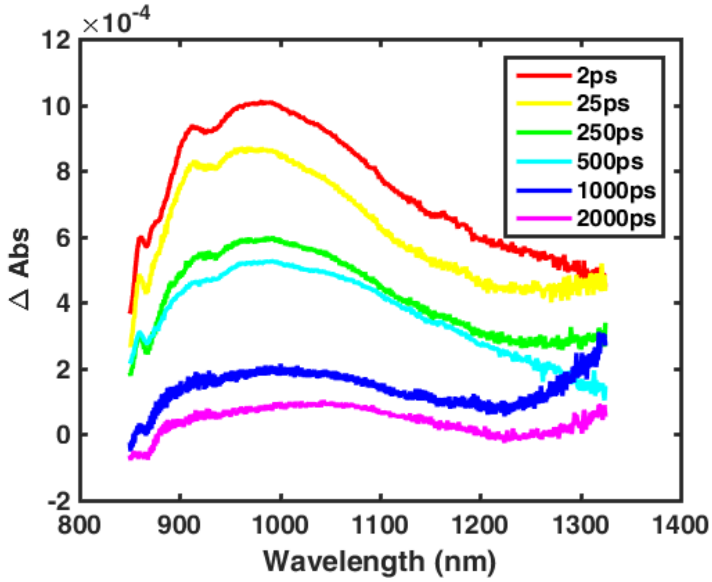

Fig S26 - Near Infrared Probe Transient Absorption spectra of **PTB7** at various time delays in chloroform with 590nm excitation. Pump power was kept at 6nJ/pulse to balance signal and minimizing annihilation events. 8 Scans were averaged. The low energy side of the spectrum past 1300nm is subject to noise due to lack of sufficient white light. The long time scans, which are spectrally distinct from the early time scans, are similar to PTB7 cation/"charge separated" features seen in previous works.[1] Data presented here, unlike that previous work is carried out in chloroform. Excited state absorption shapes have been shown to vary in different solvents in similar polymers[2], which may explain the blue shifted spectra seen here, in comparison with past reports[3].

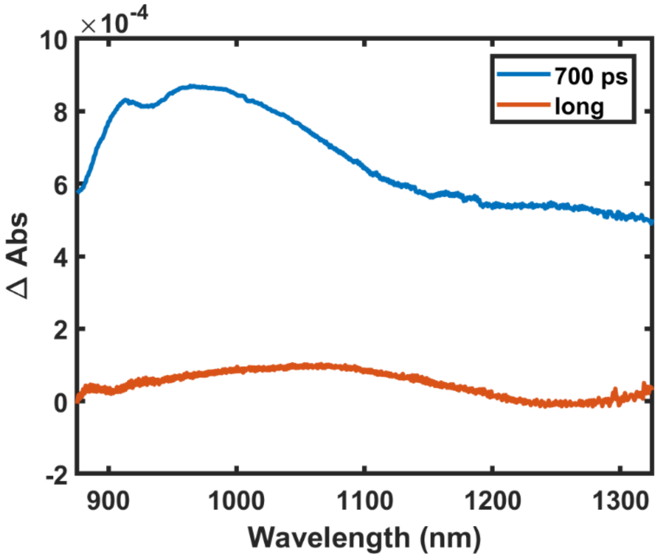

Fig S27- Decay Associated Fitting for above Near Infrared Probe Transient Absorption spectra of PTB7. The two time components agree with those seen in the visible, indicating that the

broad, 700 ps component is the exciton excited state absorption, while the long lived component is the charge separated state. An important note is that the was substantial noise red of 1300 nm, where short time kinetics had been observed in previous studies. That short time component may exist beneath the noise level of this experiment. Combined with the visible spectra, it is clear that there is no relaxation to the ground state at early times, though there maybe be interconversion of excited species.

Fit Parameter	Value
MSE	6.7416e-09
tau_1	695.5226 +- 3.4004e+01
tau_2	Long
t0	0.0379 +- 7.6481e-03
irf	0.2830 +- 2.5222e-02

Table S14 - Fitting Parameters for above fit. Tau 2 is nonvarying and not a fitting parameter.

Global Analysis Details

The data were fit globally to a sum of 2 exponentials in all cases. The dataset was then deconvoluted with the resultant intensities to reconstruct the decay associated spectra. Each wavelength is given an initial amplitude representative of its intensity at time zero. Each exponential is convoluted with a Gaussian instrument response function (IRF) with a full width half maximum as output in the fitting in the SI. The Gaussian IRF is described below:

$$IRF(t) = \left(\frac{1}{\sigma\sqrt{2\pi}} e^{\frac{-(t-t_0)^2}{2\sigma^2}} \right)$$

Where Full Width at Half Maximum for IRF=~2.355σ. Spectra were fit at multiple wavelengths throughout the spectral range. Decay Associated Spectra as shown in the main text and SI are plots of A(λ,0), each of which decays with the time constant as listed in the legend. IRF was treated as a fitting parameter, but was compared with solvent response scans to check reasonability.
